# Palliative care for people with substance use disorder and multiple problems: a qualitative study on experiences of patients and proxies

**DOI:** 10.1186/s12904-019-0443-4

**Published:** 2019-07-12

**Authors:** Anne Ebenau, Boukje Dijkstra, Chantal ter Huurne, Jeroen Hasselaar, Kris Vissers, Marieke Groot

**Affiliations:** 1 Department of Anaesthesiology Pain and Palliative Care Expertise Centre for Palliative Care, Internal Post 549, Radboud University Medical Centre (Radboudumc), P.O. Box 9101, 6500 HB Nijmegen, The Netherlands; 2 Nijmegen Institute for Scientist-Practitioners in Addiction (NISPA), Radboud Universiteit, Postbus 9104, 6500 HE Nijmegen, The Netherlands; 3 Salvation Army, Central Netherlands, Zandvoortweg 211, 3741 BE Baarn, The Netherlands; 4 Tactus Addiction Care, Location Ripperdastraat, Ripperdastraat 8, 7511 JR Enschede, The Netherlands

**Keywords:** Qualitative study, Palliative care, End-of-life, Terminal care, Substance use disorder, Addiction, Patients, Proxies

## Abstract

**Background:**

Systematic research into palliative care (PC) for people with substance use disorder (SUD) and multiple problems is scarce. The existing literature shows problems in the organizational structure of this care, e.g., lack of clear care pathways. Furthermore, negative attitudes of healthcare professionals (HCPs) and stigmatization surrounding SUD, and patients’ care-avoidance and non-disclosure of substance use are hindering factors in providing timely and person-centered PC. Furthermore, the experiences and needs of patients and proxies themselves are unknown. Therefore, this study aims to explore which problems and needs patients with SUD and multiple problems, and their proxies, experience in a PC phase.

**Methods:**

Data-collection of this qualitative study consisted of semi-structured interviews with patients with SUD and multiple problems in a PC phase, and their proxies, about their experiences in PC and their well-being. Interviews were inductively analyzed.

**Results:**

Nine patients and three proxies were included. Six patients suffered from COPD, one patient from cirrhosis of the liver and two patients from both. Seven patients stayed in a nursing home and two had a room in either a social care service (hostel) or an assisted living home where medical care was provided. Five themes were identified: 1) healthcare delivery (including HCPs behaviour and values); 2) end-of-life (EOL) preferences (mostly concerning only the individual patient and the ‘here-and-the-now’); 3) multidimensional problems; 4) coping (active and passive) and; 5) closed communication. Proxies’ experiences with healthcare differed. Emotionally, they were all burdened by their histories with the patients.

**Conclusions:**

This study shows that talking about and anticipating on PC with this patient-group appears hard due to patients’ closed and avoiding communication. Furthermore, some of patients’ EOL-preferences and needs, and coping-strategies, seem to differ from the more generally-accepted ideas and practices. Therefore, educating HCPs in communicating with this patient-group, is needed.

**Electronic supplementary material:**

The online version of this article (10.1186/s12904-019-0443-4) contains supplementary material, which is available to authorized users.

## Background

Substance use disorder (SUD) is a psychiatric disorder, which is, among other things, defined by impaired control and risky use of substances [1]. Often, it is accompanied by other psychiatric disorders and results in multiple problems on several life-domains, e.g., homelessness, unemployment or vulnerable social networks [2–6]. Although most people will recover from SUD, some remain dependent their entire lives [7, 8]. People with SUD have an increased risk of developing chronic or life-threatening conditions and of mortality [9–15] and, thus, might be in need for palliative care (PC). It is surprising, however, that systematic research on such care for this patient-group is absent and that the size of this patient-group is hardly known [16–18].

PC is an already difficult subject to investigate due to taboo on death and dying [19]. Even more challenging might be research into PC for people with SUD and multiple problems (SUD+) due to 1) patients’ tendency for avoidance coping and distrust in healthcare professionals (HCPs) [20–23]; 2) cognitive impairment and intellectual disabilities in this patient-group and; 3) feelings of shame about SUD [22, 24, 25]. Stigma surrounding SUD and particular diseases, such as COPD, might have restrained conducting research into this patient-group at all [21, 22, 26–29]. Exclusion from research, however, could mean that healthcare needs of patients with SUD+ are under-recognized [30].

The literature that is available about PC for people with SUD shows that knowledge about, and financial resources and clear pathways for this patient-group are lacking. Furthermore, this care is threatened by negative attitudes of HCPs and stigmatization surrounding SUD as well as patients’ non-compliance to treatment, their symptom representation and non-disclosure of substance use. Consequentially, physical symptoms, such as pain, are often undertreated and medical treatment and identification of PC needs appear inefficient [22, 31–41].

Despite of these barriers and the lack of well-sounded knowledge and in light of the importance of person-centered care, this qualitative study aims to explore which problems and needs, patients with SUD+, and their proxies, experience with regard to PC.

## Methods

### Study-design

A qualitative design was used, consisting of semi-structured interviews with patients with SUD+ and their proxies. Data-collection lasted from August 2017 till January 2018. For this publication, the Additional file 1: COREQ checklist (COnsolidated criteria for REporting Qualitative research) is used [42]. Previously, an extensive description of the study-design has been published [18].

### Inclusion and exclusion

To reach saturation within the qualitative data, we aimed to include ten to fifteen patients and five to ten proxies. If insufficient, more respondents would be recruited. The patients were recruited by HCPs who were specialized in SUD and had, at least, generic knowledge of PC. Proxies were approached via the patient. The main researcher (AE) included the patients based on the inclusion criteria (Table 1). AE and the patients did not have a prior relationship with each other.

**Table 1:** Inclusion criteria

Inclusion was possible if the patient: 1) was either officially diagnosed with the DSM-V classification severe ‘substance use disorder’ or informally assessed as such. A patient could either be still an active user, recently quit or in remission of alcohol, cannabis, cocaine, opioids (including heroin), sedatives and/or gamma-hydroxybutyric acid (GHB); 2) had a serious non-reversible, life-threatening somatic disease or was suffering from progressive, severe physical deterioration as a result of active addictive behaviour without the prospect of cure; 3) was 18 years or older; 4) had mastered Dutch in such way that it allowed him/her to participate in an interview; 5) was cognitively capable enough to answer interview questions (due to SUD, many patients are cognitively damaged); 6) understood what the study meant for him/her. Furthermore, the recruiting professional caregiver: 7) had to answer the following question with ‘no’: “would it surprise you if this patient would die within five years?” [43]; 8) had explicitly communicated with the patient about the fact that (s) he was not going to be cured and now reached a palliative phase.	

### Data-collection

Together with experts-by-experience (people with lived experience with SUD), the project-group developed an interview guide suitable for this patient group. Simple terminology and short questions were included. With regard to potential communication difficulties, the interviewer also brought so-called association cards to the interviews which could be used if patients would struggle to find words that covered their experiences. Furthermore, keeping into account patients’ vulnerability, the time was limited to one hour and the interviewer frequently asked for patients’ well-being [18].

Before the interview started, proxies and patients were asked for a few demographics. Semi-structured face-to-face, audio-taped interviews with patients and proxies were held by AE (experienced interviewer) at the respondents’ residents. This method enabled the interviewer to discover the respondent’ experiences. Respondents in turn, had room to explicate themselves [43, 44]. Respondents could bring somebody to the interview, but this did not occur.

Topics of the interview-guide were based on the main research question (What are the problems and needs of patients with SUD and multiple problems, and their proxies, in a PC phase?), literature and the project-group’s expertise: researchers, nurses, a manager and experts-by-experience. Topics concerned patients’ 1) needs and experiences in professional healthcare and; 2) well-being and needs on all life-domains. Proxies answered a few extra questions. The impact of SUD and the attention of HCPs to the needs of patients and proxies were related to every topic (Table 2). Proxies answered the questions from topic 1 till 4 about the patient's situation. The guides were tested within the project-group. AE made field notes during and after the interviews. Respondents were sent the verbatim interview transcripts.

**Table 2:** Main and subtopics of the semi-structured interviews

Main topics	Subtopics	
* Proxies answered the questions from topic 1 till 4 about the patient’s situation.		
1. Needs and experiences in professional healthcare	a. Care network b. Organization of care c. Communication with HCPs	Impact of SUD Attention by HCPs
2. Physical well-being and needs	a. Pain (control) b. Symptom (burden)	
3. Social well-being and needs	a. Social network/isolation b. Communication with others	
4. Psychological and existential well-being and needs	a. Life values b. Sources of strength c. Future d. Place of death	
5. Proxy experiences	a. Contact between HCPs and proxy b. Care for proxy c. Involvement in planning and decision-making d. Psychological and existential needs	

### Data-analysis

Data-analysis consisted of several steps. Grounded theory elements for inductive analysis were used by the researchers (AE and MG). To grasp informants’ meanings and experiences, first, AE and MG applied in-vivo codes to original interview text segments. They stayed close to the data to prevent over-interpretation. Since intercoder agreement between AE and MG on the first interview was high, the second and third interview were coded by AE alone [45]. MG listened to one more interview to further familiarize herself with the data and to agree on the codes attached by AE. Second, AE clustered these in-vivo together into sub-codes, which were headed under a main-code, belonging to a theme. Eventually, five themes were identified. Connections between sub- and main-codes were described and pictured in a network (axial coding). Afterwards, sub-codes and themes were supplied with a description. In this way, a code tree was generated which served to code and analyse the remaining interviews (selective coding). AE discussed the codebook with MG. When new codes or information emerged, descriptions were updated (constant comparative method) [46, 47].

After coding six interviews with the codebook, data saturation was reached; no new codes came up and adaptations to descriptions were minimal [48]. The remaining interviews were used to confirm data saturation and supply examples. Data-analysis was supported by the research software ATLAS.ti 8.0.34. To minimize researcher bias and to maximize validity, AE and MG frequently joined for discussions about, e.g., ambiguous text fragments. No member-check was executed due to time-restraints.

## Results

### Patient and proxy characteristics

During recruitment, thirty patients were found potentially eligible for inclusion, of whom nine were eventually included. Others were excluded, because they: a) denied disease (*n* = 7); b) were too ill to be interviewed or died before the interview took place (*n* = 7); c) were not willing to talk about the subject (*n* = 3); d) cancelled or denied participation for unknown reasons (*n* = 2) or; e) did not trust what would happen to their data (*n* = 2). Table 3 shows respondents’ characteristics in a narrative and anonymous matter (no gender and individual age). Patients (eight male, one female) were 61 years on average and often finished lower education and were single. None of the patients stayed in their own homes; seven stayed in a nursing home and two had a room in either a social care service (hostel) or an assisted living home where medical care was provided. One respondent was suffering from progressive, severe physical deterioration (cirrhosis of the liver) as a result of active addictive behaviour. The others had a serious non-reversible, life-threatening disease (COPD) or both COPD and cirrhosis of the liver. Only three proxies were interviewed, because either patients did not have close proxies or AE, based on the interview story, found asking for proxy participation inappropriate.

**Table 3:** Patient and proxy characteristics

Patients	
*G-01*	Diagnosed with COPD, epilepsy and arthrosis. Also G-01 had pain, and anxiety about dying. G-01 had few family connections and lost all friends. For the patient’s social life, the staff was important. The patient’s brother and spouse brought visits. G-01’s regretted how G-01 treated the spouse. Now that pain medication was effective, using cannabis became unnecessary. G-01 also used cocaine for five years, but regretted this. G-01 finished elementary school and had been homeless for three years. This interview lasted one hour and 25 min.
*G-02*	Diagnosed with COPD, cirrhosis of the liver and chronic hepatitis C. Also G-02 was suffering from cachexia, OCD and pain. G-02 was single and reunited with the own children. This patient was on friendly terms with co-patients. G-02 was still dependent on Methadone and used high doses of pain medication. This patient stated to be “addicted to addictions” and to had “used everything” in the past. G-02 finished extended primary education and mentioned a criminal history. Two interviews of 1,5 h were held.
*G-03*	G-03 had COPD and visited kidney dialysis. Also G-03 suffered from pain and sleeping and stress disorders. This patient was single and in touch with siblings, however, kept them on distance. G-02 stated to need sedatives as co-patients are found to be very annoying. Also G-03 still used much pain medication. This patient used to drink alcohol “as a habit”. Nowadays, G-03 would not state to be an addict, although thought this was actually the case. G-03 finished higher education. Two interviews of 1,5 h approximately were held.
*G-04*	This patient is diagnosed with COPD and scared of suffocation. G-04 was single and lost many friends, since disease, however was still in touch with the own mother. G-04 was on Methadone and still used weed every other day. G-04 used heroin to switch off mental pain, however, this was not needed anymore. Furthermore, this patient tried coke and LSD. G-04 did not think to be addicted anymore. This respondent finished extended primary education and had been homeless for ten years. Interviewing lasted 70 min.
*G-05*	Diagnosed with COPD, heart failure and bipolar disorder. Also G-05 had anemia and arthrosis. G-05 spent much of the time laying on bed, being in pain. This patient was divorced, but was in touch again with children and sister. Currently G-05 used painkillers and sedatives on which G-05 stated to be addicted out of necessity. In the past, G-05 used cocaine and cannabis and was “heavily addicted”. G-05 finished extended primary education and had been homeless for ten years. The interview lasted 28 min.
*G-06*	Diagnosed with COPD, chronic hepatitis C, chronic aspergilloma and underweight. This patient is single and barely had friends or family, but was well acquainted with people from the local pub. G-06 still used alcohol and Methadone and, few times a month, cocaine. In the past G-06 used heroine and many other substances. G-06 was not ashamed to still being addicted. G-06 finished extended primary education, was homeless for ten years and stated to had been in trouble with the law a few times. The interview lasted 63 min.
*G-07*	G-07 had COPD and cirrhosis of the liver. G-07 lay in bed most of the time. This patient had psychological problems, but no official diagnosis. G-07 was single and had no social network, except for a mentor. Nowadays, G-07 still used cannabis, hash and beer and stated to “never get rid” of this. Previously, G-07 also used heroin and opiates. This patient finished extended primary education. The interview was impeded and lasted only 35 min as G-07 only just met the inclusion criteria: talking was unclear as there appeared to be cognitive damage.
*G-08*	G-08 had COPD and was undernourished and in pain. Furthermore, G-08 was restless, sad, anxious and stated to hear voices. G-08 regretted to have moved to the current place. G-08 was single and had one befriended co-patient and one son, but they did not see each other much. In the past, G-08 used opiates and recently quit using alcohol. This patient stated to be still addicted to medication. G-08 finished extended primary education. This interview took 35 min.
*G-09*	This respondent was diagnosed with cirrhosis of the liver and was in much emotional pain about the past. G-09 was married and in touch with some of the foster and grandchildren. For them, G-09 wanted to stop using alcohol. G-09 stated to be still addicted. This patient finished higher education. This interview lasted over hundred minutes.
Proxies	
*H-01* *H-02*	These proxies were a child and a patient’s ex-partner. The patient could not be interviewed as a result of being cognitively and verbally disabled by Korsakov. The patient was admitted to a nursing home and was suffering from cancer of the throat with metastases. Furthermore, the patient seemed to be emotionally down, and, as tremors were worsening, seemed to be in pain. Also the patient was increasingly short of breath and needed much bed rest. The patient’s social life was limited. The patient used much alcohol, but, nowadays stopped drinking. The proxies would not call the patient addicted anymore. The proxies provided practical and emotional support. Despite having a complex history with the parent, H-02 found peace. The ex-partner, H-01, was more emotional distant. The interview lasted over hundred minutes.
*H-03*	This proxy was in emotional pain and needed to learn how to cope with the past experiences with the patient (H-03’s spouse) and how to redesign their relation. The spouse, now, finally expressed regrets of neglecting and abusing H-03. This respondent wanted to tell “the other side of the story” and stated to need to talk and ventilate. The interview lasted 143 min.

Five themes were derived from interviewing: 1) healthcare delivery; 2) end-of-life (EOL) preferences; 3) multidimensional problems; 4) coping and; 5) closed communication. The fourth and fifth theme do not answer the main question directly, but were considered important by the authors as these could impact communication during PC. Theme 4 is based on the interviews’ content. Theme 5 is based on the experiences of AE during the interviews, preparatory conversations with HCPs and proxy interviews.

### Healthcare delivery

Some patients had strong critical opinions about how they desired to be treated and which care they would like to receive. Other patients, however, were much less critical and sometimes also more grateful for the care they received. One patient said he should “have a look in the mirror first” before he could criticize care or HCPs. Also in light of great work pressure of HCPs, patients took upon a humble attitude. Furthermore, in contrast to bad experiences before, these less demanding patients stayed in places where they felt quite comfortable and taken seriously. Despite that respondents varied in their experiences and appreciations of care, some behavioural issues of HCPs and values were important in healthcare to most patients and their proxies. Quotes are presented in Table 4.

**Table 4:** Exemplary quotes

Theme and codes		Exemplary quote
1. Healthcare delivery		
*Behaviour*		
Personal attention	Q1	**“Do you have a special relation with one of the healthcare professionals?** Yes, the departments’ ‘mother’ […] she’s really empathic. When she starts her shift, first she visits me and wants to know how I’m doing. [..] just giving somebody a bit more attention than everybody else. **What does that mean to you?** That supports me.” *- G-01*
Cooperation	Q2	“The thing I’m worried about is: how will it enrol from the moment I’ll receive palliative care? [..] How would people be called in if it would happen at night, if suddenly I would become very ill? I’m afraid that everything I’ve arranged, would be for nothing. That would be a disaster. It’s no exception that healthcare professionals talk at cross purposes.” - *G-04*
Involvement	Q3	**“**At home, he distorted the truth or exaggerated his disease. I still want to talk with that doctor and know ‘how ill is he?’. I’m being left out by everyone. **Would you say the medical staff goes along with him too much?** Indeed.” - *H-03*
*Values*		
Being treated as a human being	Q4	“Well. It’s all zero. I mean. I’m here to die. And they [HCPs] highlight that, which isn’t nice, but well... You can see: even my bed is not made. Those are small things, but they sting.” *- G-02*
Patient centeredness	Q5a	“He [nursing home physician] doesn’t visit me often [..]. He goes his own way. If I want different medication, he is like ‘no’. [..] I can barely reach a compromise. To get something done, you have to manipulate a bit.” - *G-05*
	Q5b	**“What do you find important in the care you receive?** Well, I find it important that they listen to me. And would actually do something useful with that, afterwards. [..] Listen, yesterday my catheter would be cleaned and cared for. Didn’t happen.” - *G-03*
Openness	Q6	“I’ve had good experiences here. It is adequate, meaning: straight-to-the-point [..] If you promise something, keep to it.” *- G-09*
Expertise	Q7	“Listen, methadone is a strong painkiller. The problem is, in the hospital they said ‘can we give that man [patient] morphine? Will it work, because, already, his dose is so high’. I’m like: how would they know, did they try themselves?” *- G-04*
2. EOL preferences		
*The current*		
Being left alone	Q8	“They let me do my own thing, leave me alone, they just let me live. Like it’s supposed to. **Let me do my own thing?** Yes, just living like I want to. I stick to the rules as they are, but you gotta be able to live the way you want, right?” - *G-04*
(seeking) closure	Q9	**“How do you look back upon life?** That’s starting now. Actually I don’t want to talk about it, because I haven’t decided upon the whole picture. I’ve been through a lot and I’ve to find out about it and why things happened.” - *G-04*
	Q9b	“I’m feeling connected [with dad] to a certain extent, but I’m not taking it home anymore and I’m not sad about it for days, like I used to. **What were you sad about?** How things went like they did. [..] To find peace with the choices he made.” - *H-02*
*Dying*		
Without suffering	Q10	“The way I’m being sickly now, I find bad enough. If it would get worse... rather not. **Are you afraid of something?** To suffer from pain and to go downhill. Actually, to become even more dependent on others.” *- G-03*
Acceleration & alleviation	Q11	**“How would you imagine that [euthanasia]?** Just euthanasia, injecting and you are gone.” *- G-08*
Place of preference	Q12	**“Did you ever think about where you prefer to die?** I enjoy it here. **Do you prefer this place?** Yes, and I’ve made it known.” *- G-06*
3. Multiple problems		
*Physical well-being*	Q13	“And she [the physician] said ‘you could maybe prolong life with a year, but that is it’. [..] *Actually, that has passed. Probably by rest and pain medication.* And the attention. *Being in bed, getting food and more frequent [family] visits.* For six months now, each visit, we say our goodbyes. Each time you are thinking ‘this could be the last time’. [..] And that’s hard. [..] The not-knowing. Knowing it’s going to be over, but at the same time, not knowing when.” - *H-01 and H-02*
*Psychological well-being*	Q14	**“Are you surrounded by other people besides your son?** No. **And how does that make you feel?** Empty. **And does that occupy your mind much?** No. [..] **How does that effect you?** You become quiet [..] I withdraw [..] I barely eat or don’t eat at all.” *- G-08*
*Social well-being*	Q15	“She [daughter] wants to visit me. Her husband comes with her and I can understand that. He can support her. **What do you mean by supporting her?** Well, that kid didn’t see me for 25 years. [..] She was two when I disappeared off the radar. She has zero memories of me […]. **And how does it make you feel to see them [children] after such a long time?** A happening. It’s beautiful.” *- G-02*
*Existential well-being*	Q16	**“And how do you look back upon life?** […] When I reflect upon it, do a sum of the good and bad things and take the mean score, I would say ‘pointless’. Useless. If I would’ve had a wife and kids, it would’ve been different. […] I don’t think I made the world a better place […]. Wouldn’t I have been here, nobody would’ve noticed.” - *G-03*
4. Coping		
*Active*		
Sources of strength	Q17	“I don’t have the age to die yet. **No.** I’m still fighting and living from one date to the next. Recently, I’m married for 25 years. **Congrats.** Thanks. The day before yesterday, I’m together with my wife for 29 years. That’s how I live on. From date to date.” *- G-01*
Seeking social support	Q18	**“And how do find strength?** In myself and other human beings. Not in religion or something like that. [..] **Could you tell me more?** Well, we [co-residents] communicate with each other. Since we are burdened with one another, we become a kind of unity. It‘s easier to struggle with your problems. [..] **What do you mean by unity?** We are in the same ship together. That’s a unity. A kind of Titanic. **With your co-residents or also with the staff?** They too, indeed.” *- G-06*
Avoidance/distraction	Q19	“I’ve still got those suicidal thoughts sometimes. **[…**] **and what does alcohol provide you with?** Alcohol gives me peace […]. It tempers, right? **Those negative feelings?** Yes.” *- G-09*
Fighting	Q20	“On the one hand I would embrace death, but body and mind think differently. I wanna get rid of the pain, but at the same time I’m fighting to live, while death is lurking.” *- G-02*
Blaming	Q21	**“And a few minutes ago I asked whether your physical state is related to the alcohol and you said ‘yes, of course’.** Yes. **What do you think of that?** It sucks. It is what it is, though.” *- G-09*
*Passive*		
Absorption	Q22	**“And shortness of breath, what does that mean to you?** I find it terrible. It’s part of the COPD, though. It sometimes reminds me of the terrible disease I’ve. But well … I’ve to learn how to live with it. **It’s confronting? If I understand it right.** Yes. **How do you cope with that?** I can’t ignore it. I’ve to wait till it’s over and keep calm.” *- G-03*
Resignation or acceptance	Q23	**“And how do look back on life?** ‘To be or not be’, you know. It’s what it is. I’ve always lived on the other side of the coin or on the edge. [..] I’ve always walked downhill, never uphill. So you should’ve resignation. I’m resigned. [..] I’m not full of self-pity and bitterness.” *- G-06*
	Q24	“I have to lie in bed all the time, smoke a cigarette. That’s all I’ve got. I’m looking forward to the moment the lights go out. I’m fine with that. I’ve been waiting for it for months now.” *- G-05*
Disinterest	Q25	“I’ve had so many conversations, but there’s a point when there’s nothing left to say. When I look forward, there is few … [silence]. **What do you mean by looking forward? You said ‘there’s not much left to say, when I look forward’?** Yeah, boredom. [..] There’s no future.” *- G-07*
5. Closed communication		
*Expression*	Q26	**“And with whom do you talk about your disease?** With nobody. With my son. I provide him with all the information I’ve got. [..]. It’s making me sick to tell it a hundred times, to repeat it every time. **How so do you find that unpleasant?** It’s exhausting.” *- G-05*
	Q27	**“And is there something you want to do about it [feeling empty]?** What should I do about it? Such bullshit! **Well yeah, I’m just being curious.** It’s nothing. **It doesn’t sound pleasant to me.** It isn’t indeed. **But you don’t want to talk about it?** No.” *- G-08*
*Disease awareness*	Q28	**“Do you have a physical disease at this moment?** As far as I know, I haven’t. **No?** No. **You are not suffering from something in your lungs, e.g. COPD?** Only methadone. **You are using methadone, okay, but you don’t have a physical disease?** No.” *- G-07*

#### Behaviour of HCPs

First, patients stated to appreciate HCPs’ personal attention, in which taking time and being genuine, were important. Especially, nurses and volunteers were mentioned as living up to this. Care, however, appeared to often focus on the medical or physical dimension due to lack of caregivers’ time (Q1). Second, only few patients mentioned they valued cooperation between HCPs, however, did not always experience this as such (Q2). Finally, proxies mentioned that they wanted to be involved in patient care. One proxy also stated that she really needed to be actively involved, because the patient sometimes lied or manipulated (Q3). Proxies differed in their opinions about the extent of shared decision-making, the support they received themselves and the access to univocal and up-to-date information.

#### Values

Being treated as a human being by HCPs was found of great importance within PC, i.e. recognition and respect for the person behind the patient and taking their needs and emotions seriously, despite their (history of) substance use. Also patients did not wish to be treated like an ill person or a child. Being treated as a human created trust and feelings of being heard. Although “the little things” could also add to this, it was not always experienced by patients (Q4).

Furthermore, interviews showed two possible ways of patient-centeredness. The first concerned a central position of the patient in care and in decision-making. Some patients with SUD+ wanted to be strongly in charge. Patients did not appear to prefer interference and rules, but instead, desired freedom and independence. However, most patients were admitted to settings where substance use was not allowed or only in controlled dosages. Although most patients stated to respect and understand this, some expressed dissatisfaction (Q5a). The second way of patient-centeredness concerned a personalized way of care giving, i.e. the extent to which care and communication between patient and HCP suited patients’ (end-of-life) preferences and needs. Experiences on such personalized care, however, differed (Q5b). Adequacy, alertness and knowing a patient were found important. Especially the latter was recognized by proxies if they acted as spokespersons of patients who were too cognitively or verbally disabled to communicate sufficiently.

Also patients appreciated straightforward communication and openness about diagnoses and care practices (Q6). Finally, some patients mentioned that HCPs who were important to them, possessed medical knowledge as well as mastered medical practice. Other patients, however, questioned such expertise. They stated to always have to be alert in order to receive the care they needed. Sometimes patients claimed to know more than their HCPs, e.g., about methadone (Q7).

### EOL-preferences

During interviewing, the involvement and willingness to talk about EOL-preferences and EOL-decisions differed greatly between patients. Also despite that the interviewer asked about the future and even afterlife, most patients spoke about desires concerning the short-term and the last days only. The period in between remained largely unspoken as patients appeared to focus on the ‘here and now’. Consequentially, EOL-preferences can be divided into ‘the current’ and ‘dying’. The extent of HCPs’ attention for these preferences and, consequentially, written or verbal agreements for future-plans varied.

#### Current EOL-preferences

Patients strongly preferred to be left alone in their EOL-phase and to keep on doing their own, often small, things, e.g., smoking cigarettes and watching television. Their own room was important to them as this was a private place, with their own stuff and way of living and some sort of freedom and independency (Q8). Most patients had (last) wishes relating to themselves only, e.g., going on trips or coping with the past (Q9a). Only one patient was working on closing a social relationship in which forgiveness played a major role. Proxies more often expressed a need for closure (Q9b).

#### Preferences for dying

Three preferences were shared among patients. First, ‘dying without suffering’, which meant dying without pain and gradual deterioration. A sudden and quick death or dying in their sleep, was preferred. Patients with COPD were anxious to suffocate. Being dependent on healthcare professionals, hindered a feeling of being in charge (Q10). Second, patients did not state explicitly that they wanted to prolong life. Instead, they more often expressed a wish to accelerate death or alleviate potential future suffering, respectively by euthanasia and palliative sedation. One patient wished to die, because he was not allowed to continue drug use. Patients had their own ideas about how euthanasia and palliative sedation would work (Q11). Others spoke of unofficial euthanasia, e.g., dying by overdose or taking saved up medication. Finally, whereas few patients did not mind about their place of death, most patients desired to die at the place they currently stayed (Q12).

Only one patient wished to have his wife around during the last days. Not wanting to die alone, was not stated explicitly by other respondents. Another subject, that was not often mentioned, concerned a feeling of being a burden to others. Only few worried about their loved ones during or after the patient’s dying. Indeed, EOL-preferences implied individual preferences more than social preferences (meaning: including others).

### Multiple problems

Previous long-term and/or present persisting substance use influenced the extent and diversity of the problems of this study’s patient-group. Patients had different experiences in HCPs’ attention for their well-being on all four dimensions of PC: physical, psychological, social and existential.

#### Physical well-being

Patients suffered from particular diseases, such as COPD or cirrhosis of the liver. Other consequences of substance use were continued dependency on methadone and craving for substances. Furthermore, being COPD-related, patients often were short of breath and anxious to suffocate. Patients mentioned that being tired and immobile, decreased the quality of life (QoL) and had consequences for social, physiological and existential well-being. Furthermore, many patients suffered from (uncontrolled) pain, despite high doses of pain medication. Although some patients were in deterioration, other patients stated to stabilize or even recover. For proxies, this came with insecurity (Q13).

#### Psychological well-being

Several patients had psychiatric co-morbidities, e.g., depression. Some patients also felt shame and regret about past issues. Furthermore, psychological well-being sometimes was affected by a limited or an absent social life (Q14). Paradoxically, feelings of being alone thus existed besides the desire to be left alone. Also proxies experienced psychological problems, mostly referring to sadness or anger caused by the history with a patient. In such case, closure could provide peace (Q9b).

#### Social well-being

Analysis shows that patients’ social lives were limited and consequentially, some patients attached much value to an alternative social network: co-residents, volunteers or healthcare staff. Broken networks could be due to history with substance use. Only few patients had friends and family, who provided practical and emotional support. The relationship between them, however, appeared distant. Proxies, for example, called themselves “volunteer”. In some cases, though, the last phase of life allowed patients and proxies to re-evaluate or rebuild their relationship (Q15).

### Existential well-being

Patients’ existential issues varied greatly. By being confronted with death and dying, patients sometimes gained insights into life or themselves and stated to be changed (positively) as a person. Furthermore, existential loneliness was implicitly expressed by some patients (Q16), whereas proxies explicitly stated so about patients.

### Coping

Patients handled their multiple problems, history with substance use, their incurable disease and approaching death, differently. Various coping styles came to surface during the interviews, which can be divided in active and passive coping styles. These changed over time and differed per situation.

#### Active coping strategies

Patients had several sources in which they found strength to feel well and cope with their circumstances, such as religion or nature (Q17). Some, but not many, also sought or received emotional support from their proxies, HCPs or volunteers (Q18). Others found that they should cope alone. Furthermore, patients tempered or tried to avoid pain and shortness of breath by using substances (Q19). Distracting and ignoring feelings or talking about EOL issues were expressed too.

Some patients also responded to their situation by “fighting”. They expressed great desire to live and stated things like “I can’t be beaten” (Q20). They indeed lived longer than they themselves or their HCPs expected. Only few patients coped by blaming their substance use for social problems and cognitive or physical well-being. The latter, however, was only explicated when AE asked for it (Q21).

#### Passive coping strategies

Some patients reacted to their situation by being overwhelmed with pain, suffering or thoughts about disease, i.e. ‘absorption’. They were unable to let go (Q22). Another way of passive coping concerned resignation or acceptance, in which down-to-earthiness played a role (Q23). Sometimes also patients’ own experiences with mortality lead to a certain acceptance of death as they “already have had many chances”. They were, however, worried about the process of dying, caused by experiences with the dying of others. Beyond acceptance was a welcoming of death as this would unburden proxies and would mean being at peace and free of pain (Q24). The last passive coping style concerned ‘disinterest’. Some patients did not feel up to invest in future-plans or emotional or physical improvement. A few patients appeared even cynical and literally stopped doing things. Attaching to life and the future and having hope were let go (Q25). Such patients seemed rather short-spoken.

### Closed communication

Going deeply into the interview topics appeared hard and sometimes was hindered by several issues. The first is expression. Patients’ answers, often, were short and closed. Some patients seemed to prefer simple questions and terminology. Others were quite avoiding in their answers or stated that they “are not feeling up” to, e.g., the interview or talking about EOL-issues. Sometimes, answers were incomprehensible, contradictory or incomplete (Q26–27). Second, during interviews, there was quite much restlessness, often caused by craving to, for example, smoke. Third, whereas patients during parts of the interviews seemed aware of their disease or their PC phase, they did not seem to be so at other moments during the interview or they stated that they did not know much about it (Q28). Proxies stated that, as a consequence of patients’ closedness in communication, they themselves were allocated bigger responsibility and a greater role within care. Also both proxies and HCPs stated that observations were important to allow for patient-centered care.

## Discussion

This study managed to explore problems and needs of patients with SUD+, and their proxies in PC even though these patients sometimes appeared to struggle with talking about and anticipating on PC trajectories due to, among other factors, avoidance coping. The results show that some patients wanted to be in charge of their care, whereas others were less critical on the care they received. The amount of time HCPs had available, was short and sometimes hindered personal attention and personalized care. Patients’ EOL-wishes did not often involve other people. Instead, most patients preferred solitude without much interference of others. This, though, existed alongside with patients’ feelings of being lonely. Their (care) needs were focused on the ‘here and now’ and the terminal care phase only. Consequentially, potential other care needs remained undiscussed. Patients stated to “fight” disease, whereas at the same time, they welcomed death. Interviews furthermore show that proxies had much emotional burden and were - sometimes out of sheer necessity - strongly involved in care. Their experiences with support from HCPs differed.

### Practice implications

This study has several practice and educational implications. First, the high number of patients who were excluded because they were too ill, might indicate non-timely identification of PC phases within this patient-group. This assumption is confirmed by other studies, in which late identification of PC needs was, among other factors, attributed to a lack of knowledge about PC in this patient-group on the one hand and patients’ care-avoidance on the other [39, 40]. Consequentially, patients might miss opportunities for prolonged survival, higher QoL and decreased symptom burden [49–52]. Therefore education about early identification of PC is needed. In light of patients’ limited communication, attention for observing, e.g., body language or changes in day-to-day functioning indicative of deteriorating health, is important [53, 54].

Second, patients’ EOL-preferences and ways of handling their disease seem somewhat different compared to the more general PC population. Findings show comparisons, for example, with regard to anxiety about suffering from pain, but also differences. Whereas other populations define ‘a good death’ by, for example, being prepared, saying goodbyes, and having family support [55, 56], this patient-group does not appear to do so necessarily or at least, not explicitly. Also the way respondents took up health and disease seems different. In general, being ill imposes patients with a certain responsibility and involvement in improving their situation, in exchange for a temporary removal of, e.g., employment [57, 58]. Not all respondents from this study, however, did fulfil this generally accepted ‘sick role’. Some even remained active users and some were not very willing and avoiding to talk about death or dying. Since patients with SUD+ thus seem to differ in their EOL-preferences, healthcare behaviour and coping, it is not unimaginable that HCPs who are unfamiliar with this patient-group might experience difficulties in understanding and discomfort in caring for this patient-group. A comparable study indicates the same practice implication and points at the importance of a non-judgmental attitude [59]. Stigma on SUD is a major issue in caring for this patient-group and education or bed-side teaching could be helpful [60].

Third, to provide person-centered care, HCPs need to know what is and will be of importance to a patient. This though is hindered by patients’ focus on the ‘here-and-the-now’ and their closed communication. This short-term focus was found in another study about homeless people in PC too [61]. Furthermore, for patients to be aware of and express what is important in their last phase of life, they need the ability to rationalize and understand their disease and death [33, 61–64]. Respondents of this study sometimes temporarily lacked such realisation. If advance care planning is to be adopted for this patient-group, HCPs thus need to create trust and have alert and enduring attitudes. Repeating as well as clear conversations about EOL preferences are important [59]. A dedicated nurse supervisor may be an option. Getting insight into life stories via proxies, furthermore, might be helpful to retrieve patients’ desires. These proxies, though, need to be supported, as was suggested in other studies too [39, 65].

### Future research

Future research into the diversity of the patient-group should be undertaken as it is likely that we interviewed a subgroup. The bigger patient-group might include patients that are cognitively or intellectually more disabled or not admitted to healthcare services. Also more as well as bereaved family caregivers could be interviewed to complement their experiences. Finally, observations of patients during entire last phases of life, may provide insight into what they find important in-between the ‘here-and-the-now’ and the final days.

### Strengths and weaknesses

This study adds much insight to the existing literature into the experiences of patients with SUD+ in a PC phase, and their proxies. Despite the well-adapted design of the interview guide and the respectful attitude of the interviewer, avoidance coping and possibly shame might have impacted the openness and duration of the interviews. Also only three proxies were included. Finally, the current sample includes only patients from in-patient setting. However, whether this patient-group is at all able to live at home because of their complex problems and lack of informal caregivers, is questionable.

## Conclusion

This study is one of the first to investigate the experiences of patients with SUD+, and their proxies, in a PC phase. Talking about and anticipating on PC appeared hard due to, among others, patients’ closed communication. EOL-preferences were focus on ‘the here and now’ and were not often related to other people. Proxies’ experiences with professional healthcare differed, but emotionally they were all burdened by their histories with and care for the patients.

Insights of this study should be taken into account in both organization of PC and the care-provision itself. Suggestions were made to improve the communication with this patient-group, the identification of a PC phase and person-centred care.

## Additional file

Additional file 1: COREQ (COnsolidated criteria for REporting Qualitative research) Checklist. (PDF 554 kb)

## Data Availability

The datasets used and/or analyzed during the current study are available from the corresponding author on reasonable request.

## Abbreviations

COPD: Chronic Obstructive Pulmonary Disease
COREQ: COnsolidated criteria for REporting Qualitative research
EOL: End-of-life
HCPs: Healthcare professionals
PC: Palliative care
QoL: Quality of life
SUD: Substance use disorder
SUD+: Substance Use Disorder and multiple problems
